# In Vitro Antagonistic Effect of Gut Bacteriota Isolated from Indigenous Honey Bees and Essential Oils against *Paenibacillus Larvae*

**DOI:** 10.3390/ijms21186736

**Published:** 2020-09-14

**Authors:** Miroslava Kačániová, Margarita Terentjeva, Jana Žiarovská, Przemysław Łukasz Kowalczewski

**Affiliations:** 1 Department of Fruit Science, Viticulture and Enology, Faculty of Horticulture and Landscape Engineering, Slovak University of Agriculture, Tr. A. Hlinku 2, 94976 Nitra, Slovakia; 2 Department of Bioenergetics, Food Analysis and Microbiology, Institute of Food Technology and Nutrition, University of Rzeszow, Cwiklinskiej 1, 35-601 Rzeszow, Poland; 3 Institute of Food and Environmental Hygiene, Faculty of Veterinary Medicine, Latvia University of Life Sciences and Technologies, K. Helmaņaiela 8, LV-3004 Jelgava, Latvia; margarita.terentjeva@llu.lv; 4 Department of Plant Genetics and Breeding, Faculty of Agrobiology and Food Resources, Slovak University of Agriculture, Tr. A. Hlinku 2, 94976 Nitra, Slovakia; jana.ziarovska@uniag.sk; 5 Department of Food Technology of Plant Origin, Poznań University of Life Sciences, 31 Wojska Polskiego St., 60-624 Poznań, Poland

**Keywords:** *Lactobacillus* spp., rectum, intestine, antimicrobial activity, antimicrobial resistance, essential oils

## Abstract

The aim of study was to isolate and identify the gut bacteria of *Apis mellifera* and to evaluate antagonistic effect of the bacteriota against *Paenibacillus larvae*, which causes American foulbrood (AFB) in honeybees. The dilution plating method was used for the quantification of selected microbial groups from digestive tract of bees, with an emphasis on the bacteriota of the bees’ intestines. Bacteria were identified using mass spectrometry (MALDI-TOF-MS Biotyper). Overall, five classes, 27 genera and 66 species of bacteria were identified. Genera *Lactobacillus* (10 species) and *Bacillus* (8 species) were the most abundant. Gram-negative bacteria were represented with 16 genera, whereas Gram-positive with 10 genera. *Delftia acidovorans* and *Escherichia coli* were the most abundant in the digestive tract of honey bee. Resistance to a selection of antimicrobials was assessed for the bacterial isolates from bee gut and confirmed against all antimicrobials included in the study, with the exception of cefepime. *Lactobacillus* spp., especially *L. kunkeei*, *L. crispatus* and *L. acidophilus*. showed the strongest antimicrobial activity against *P. larvae*, the causal pathogen of AFB. Antimicrobial activity of essential oils against isolated bacteria and two isolates of *P. larvae* were assessed. Application of a broad selection of plant essential oils indicated that *Thymus vulgaris* had the highest antimicrobial activity against *P. larvae*.

## 1. Introduction

The digestive tract of the worker bee is inhabited with a variety of microorganisms diverse in their morphology, physiology and metabolism. The microbiota of digestive tract consists of yeasts (1%), Gram-positive bacteria (29%) and Gram-negative and gram-variable bacteria (70%) [1]. The first research on microbiota of digestive tract of bees had been published in the beginning of the 20th century and *Lactobacillus rigidus apis, Lactobacillus constellatus* and *Bacillus influenzoides apis* were found the main representatives of digestive tract microbiota. Subsequent reports on microflora studies of bees and microorganisms in their diet were published in the 1960s [2,3]. It has been agreed that the only probiotic bacteria species present belonged to Bifidobacteria [4].

American foulbrood (AFB) is a disease caused by aerobic to microaerophilic, Gram-positive, spore-forming rod, *Paenibacillus larvae*. The disease causes huge economic losses to beekeepers around the world [5]. *P. larvae* affects honey bee larvae in period when it takes food, rendering the bee larvae more susceptible between 12 to 48 h of life. Bacterial spores germinate in the gut of larvae, bacteria multiply and kill the larvae at pre-pupal or pupal stage. Infected larvae are settled at the bottom of the cells with sunken sealed brood appearance. The disease is highly contagious as more than 2.5 billion oval spores could be produced in 10 days. AFB does not affect the adult bees, but they facilitate the spread of infection within a colony [6].

The use of antimicrobials in beekeeping is permitted in the United States and is also used in South America and some East Asian countries. In the European Union, the application of antimicrobials in beekeeping is banned in some countries [7].

In recent years, there has been a growing interest in application of natural substances, including for pathogen and pest control: chemical compounds of plant secondary metabolism, extracts or vegetable oils supporting green consumer behavior and healthy lifestyles trends. The diversity of plants stimulates the search and research of new plant-based chemical compounds. Some of identified compounds share antimicrobial activity against pathogenic microorganisms and have even appeared in controlled clinical trials [8]. In particular, the essential oils and mixtures of mono- and sesquiterpenes are known for their strong antimicrobial activity. The possible applications include, inter alia, food production, medicines or cosmetics industries [9].

Therefore, the aims of this study were: (i) to isolate and identify bacteria from the digestive tract of adult honeybee workers (*Apis mellifera*), (ii) evaluate the antagonistic effects of selected bacteria from the bee gut against the bacteria *P. larvae* and (iii) detect antimicrobial activity of essential oils against *P. larvae*.

## 2. Results

### 2.1. Bacteriota of Adult Worker Bees (Apis mellifera)

Groups of bacteria isolated from the digestive tract of summer and winter adult worker bees are shown in Table 1. The highest counts of aerobic microorganisms were found in the intestine of winter bees (5.39 ± 0.14 log cfu/g) and the lowest in the rectum of summer bees (4.48 ± 0.13 log cfu/g). The total counts of anaerobic microorganisms ranged from 8.12 ± 0.06 in the intestine of summer bees to 9.25 ± 0.15 log cfu/g in the rectum of winter bees. Anaerobic Gram-positive microorganisms counts ranged from 6.13 ± 0.09 for summer bees in the intestine to 7.10 ± 0.12 log cfu/g for winter bees in the rectum. The lowest counts of *Bacillus* spp. were found in the intestine of winter bees (2.48 ± 0.09 log cfu/g) and the highest were found in the winter bees in the rectum (3.53 ± 0.07 log cfu/g). The lowest counts of *Lactobacillus* spp. were found in the intestine of winter bees (7.14 ± 0.06) whereas the highest were found in the rectum of winter bees (8.27 ± 0.11). The coliform bacteria counts were the highest in the rectum of the winter bees (3.57 ± 0.13) whereas the lowest counts were in the intestines of the winter bees (2.52 ± 0.11). There were statistically significant differences among all groups of microorganisms (*p* ≤ 0.05, *p* ≤ 0.01).

### 2.2. Isolated Bacteria from Bees Gut

A total of five classes of bacteria were obtained from the gut of the honey bee: Actinobacteria, Alphaproteobacteria, Betaproteobacteria, Firmicutes and Gammaproteobacteria. A total of 27 genera were isolated from the honey bee bacteriota: *Aeromonas, Arthrobacter, Bacillus, Citrobacter, Delftia, Enterobacter, Enterococcus, Escherichia, Fructobacillus, Hafnia, Klebsiella, Kocuria, Lactobacillus, Lactococcus, Microbacterium, Moraxella, Morganella, Paenibacillus, Pantotea, Proteus, Pseudomonas, Rahnella, Ralstonia, Raoultella, Serratia, Sphingomonas* and *Staphylococcus*. A total of 66 species were isolated from bees, of which the genus *Lactobacillus* represented by 10 species and the genus *Bacillus* by eight species were the most numerous (Table 2).

**Figure 1:**
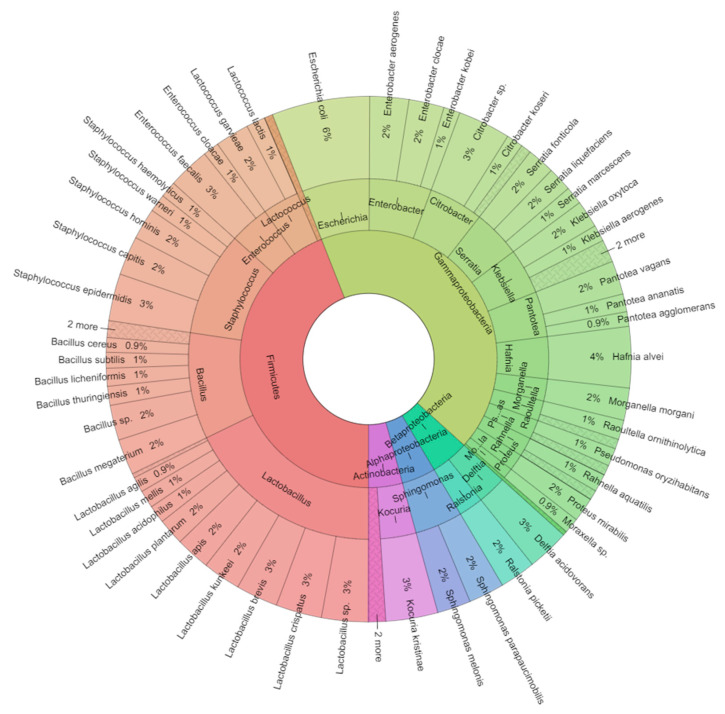
Krona RSF display of total bacteriota isolated from bee digestive tracts. Presented are the frequencies of detected species, genera and classes, from the outer ring inwards.

**Figure 2:**
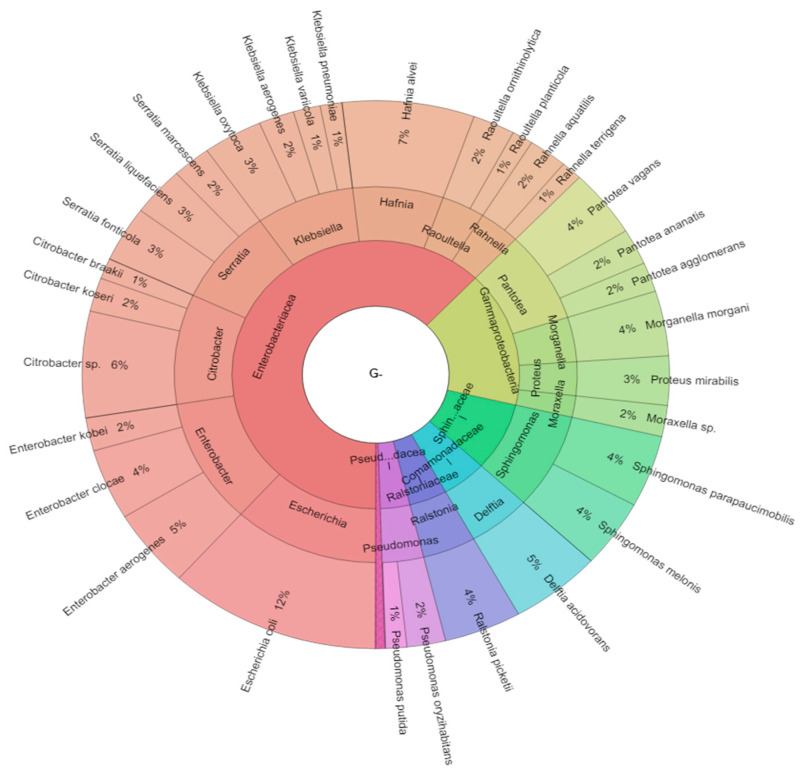
Krona RSF display of Gram-negative bacteriota isolated from bee digestive tracts. Presented are the frequencies of detected species, genera and classes, from the outer ring inwards.

**Figure 3:**
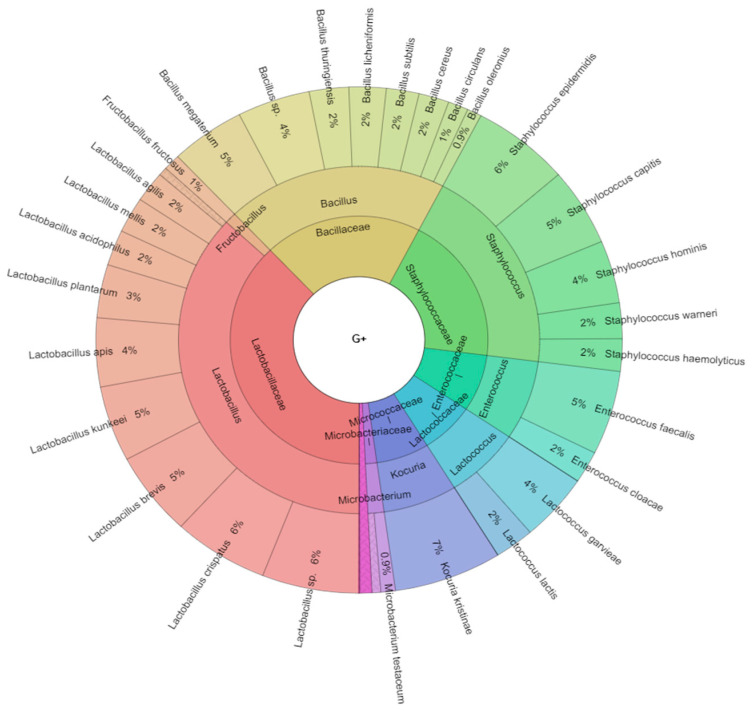
Krona RSF display of Gram-positive bacteriota isolated from bee digestive tracts. Presented are the frequencies of detected species, genera and classes, from the outer ring inwards.

In total, there were 10 genera of the Gram-positive and 16 genera of the Gram-negative bacteria isolated in the study. MALDI-TOF-MS Biotyper identification score for *Lactococcus garvieae* ranged from 2.015 to 2.026, *Kocuria kristinae* from 2.035 to 2.563, *Staphylococcus capitis* from 2.035 to 2.503, *Staphylococcus epidermidis* from 2.050 to 2.445, *Staphylococcus hemolyticus* from 2.041 to 2.341, *Staphylococcus hominis* from 2.150 to 2.345, *Staphylococcus warneri* from 2.053 to 2.545, *Hafnia alvei* from 2.296 to 2.563, *Morganella morganii* from 2.198 to 2.578, *Pantoea ananatis* from 2.196 to 2.363, *Pantoea agglomerans* ranged 2.371 to 2.466, *Raoultella ornithinolytica* from 2.051 to 2.550, *Raoultella planticola* from 2.198 to 2.428 and *Serratia fonticola* from 2.190 to 2.251, indicating reliable identification of bacterial species. Similarly, high scores were achieved for the other identified species. From the taxonomic point of view, 42.8% of isolates belonged to the class Gammaproteobacteria, whereas 43.9% to Firmicutes, 4.8% to Betaproteobacteria, 4.3% to Actinobacteria and 4.2% to the class Alphaproteobacteria (Figure 1, Figure 2 and Figure 3). Isolates of Gram-negative bacteria belonged to the families Aeromonadaceae, Comamonadaceae, Enterobacteriaceae, Pseudomonadaceae, Ralstoniaceae and Sphingomonadaceae of *Proteobacteria phylum*. Gram-positive bacteria belonged to the families of Bacillaceae, Enterococcaceae, Lactobacillaceae, Lactococcaceae, Microbacteriaceae, Micrococcaceae, Paenibacillaceae, Staphylococcaceae of phyla Actinobacteria and Firmicutes.

A total of 66 species of bacteria from the digestive tract of bees were isolated, of which 33 were Gram-positive and 33 Gram-negative. *Escherichia coli* was isolated most frequently from all samples tested, but *P. larvae* was isolated from only one sample (Table 3).

### 2.3. Antibiotic Resistance of A. mellifera Gut Bacteriota

A total of 5789 isolates were isolated from the digestive tract of 200 bees. Gram-positive and Gram-negative bacteria showed antimicrobial resistance to various classes of antimicrobials (Table 4).

### 2.4. Antimicrobial Activity of Isolated Bee Digestive Tract Bacteriome against P. larvae

The interactions between intestinal bacteria and pathogens of *A. mellifera*, in particular the action of intestinal bacteria against *P. larvae*, are an area of great research interest. Research on microbial composition of digestive tract of *A. mellifera* are perspective from the bee’s health point of view. The research on antagonisms of *P. larvae* may promote the development of bee-friendly compounds, to protect the bees from infection with pathogens.

All microorganisms tested showed antimicrobial activity against *P. larvae*. The strongest antimicrobial activity was shown by *Lactobacillus*, whereas the weakest was typical for Enterobacteriaceae (Table 5). Among the species analyzed, *L. kunkei, L. crispatus, L. acidophilus* were the most active against *P. larvae*. *Klebsiella variicola, Ralstonia picketii, Pantotea agglomerans, Pa. vagans* and *Serratia liquefaciens* were less active against *P. larvae* isolated from bee intestines. The strongest antimicrobial activity of *L. kunkei, L. acidophilus* and *L. crispatus* and the weakest antimicrobial activity of *Pa. ananatis* and *Rahnella aquatilis* were found against *P. larvae* CCM 4483.

### 2.5. Antimicrobial Activity of Essential Oils against P. larvae

The next aim of the work was to determine the antimicrobial activity of essential oils against two strains of *P. larvae*. The highest antimicrobial activity (Table 6) was recorded for *Thymus vulgaris* (19.67 ± 1.53 mm and 15.67 ± 1.53), *Origanum vulgare* (18.67 ± 1.15 and 19.00 ± 1.00 mm, respectively) and *Pinus montana* (17.67 ± 0.58 and 17.33 ± 0.58 mm, respectively). The lowest antimicrobial activity was recorded for *Citrus sinensis* (2.00 ± 1.00 mm).

## 3. Discussion

The highest counts of the aerobic microorganisms, *Bacillus* spp., *Lactobacillus* spp. and coliform bacteria were found in the intestine of winter bees and the lowest in the rectum of summer bees. Similar results of bacterial counts have been reported previously [10,11,12,13]. The microbiome of bees represents not only the microorganisms present in the adult worker bees, but also reflects the hive microbiota. The origin of hive microorganisms are nectar, pollen, dust and other airborne and soilborne environmental contaminants [12,13,14]. The excrement of honey bees and animals could be a source of microbiota during nectar harvesting. A wide variation in bacteria associated with bees have been ascribed to the external environment [15]. The bacteriota of the digestive tract of the Japanese eastern bee (*Apis cerana japonica*) revealed that *Bacillus* species could be potential antagonists for biologic control of *P. larvae* [16].

Non-culture studies of bee microbiome were conducted on the digestive tract or only on the middle and posterior parts of the intestines [17,18,19,20,21,22,23,24,25] and revealed that the pollination-based environmental microbiota and the four nectar-bearing ones are an important source of the beneficiary and potentially beneficiary microorganisms for bees [26,27,28]. *Lactobacillus* spp. were frequently found in the bee intestines and were considered the most important genus of lactic acid bacteria (LAB) in promoting animal and human health [11,29,30,31]. *Lactobacillus* spp. play significant role in feed digestibility in animals and they are important for functioning of gastrointestinal tract and accompanied immunological responses [32,33,34,35,36,37]. In our study, we did not identify species from the *Bifidobacterium* genus.

Antimicrobial resistance of the bacterial isolates varied in our study, depending on the genus and strain properties. Kačániová et al. [38] found resistance to tigecycline (12.5%) and amikacin (18.2%), gentamicin (9.5%) and chloramphenicol (7.2%) in their bacteriome of honey bees. Administration of antimicrobials triggers changes in the microbiome of humans and livestock, therefore, assessment of the effect of the antimicrobials on bee intestinal microorganisms is important for their health prognosis [23,24,39,40] and a possible explanation of unexpected bee colony deaths [41]. The studies on microbiome diversity and its antimicrobial resistance can provide an overview on nutritional and health problems of honey bees [42].

American foulbrood (AFB) is the most destructive bacterial disease of honey bee larvae [43]. AFB is a contagious infection that begins in an individual bee larva and can cause the collapse of the entire colony because only a few spores of *P. larvae* are necessary to initiate the disease [44].

The use of antimicrobials, especially oxytetracycline, could protect the bees hives against infection, however, *P. larvae* resistance to oxytetracycline has been identified in the USA, Argentina and Canada [5,45]. Use of antimicrobials in beekeeping poses a serious risk to human health as their residues may persist in honey and other bee products [46]. Adverse effects of application of antimicrobials on the honey of honey bees [47] and on the beneficial intestinal bacteria [48] have been described.

The biologic control of AFB pathogen is considered an environmentally conscious and bee-friendly perspective. Evans and Armstrong [49,50] found that certain intestinal bacteria of *A. mellifera* showed antagonistic activity against *P. larvae*. Eastern Japanese bee (*Apis cerana japonica*), native to Japan, exhibited resistance against parasitic and microbial pathogens, including mite and AFB pathogen [51]. The antagonistic effect of bacteria may also depend on bacterial communities present or strains properties, including production of antimicrobial substances, e.g., bacteriocins and lysozyme and changes in pH as a result of organic acids production [52]. Bacteria with antagonistic properties enhance control or inhibition of pathogens. *Bacillus* spp. were found to exhibit bactericidal and fungicidal effects in the host gut as a result of production of various antimicrobial compounds [53,54]. *Apis mellifera jemenitica* was shown as biologically better adapted to harsh environment with higher productivity [55,56].

Several natural compounds were studied for antagonistic activity against *P. larvae* in vitro [57,58,59], however, the identified cytotoxic effects on bees had limited their practical application. Alternatives, such as prevention and control methods of the AFB pathogen are an area of great interest. Since the ancient times, the herbal medicine and herbal extracts were applied for treatment of human and animal diseases [60]. Biologically active compounds of honey, propolis, essential oils, agents from spore of bacteria of honey and fungal extract of pollen were tested against AFB pathogen [61,62,63,64,65]. Of these, essential oils showed the strongest antibacterial activity against microorganisms responsible for bee diseases without toxicity on bees in vitro. The main complication in those studies is to obtain the results applicable to beekeeping related to the antimicrobial activity of essential oils and their effect on bees [66,67]. In our study, *Thymus vulgaris* was the most effective essential oil against both species of *P. larvae*, whereas the most effective essential oils against *P. larvae* CCM4483 were those from *Pinus silvestris* and *Abies alba*.

Tests of *Melaleuca viridiflora* and *Cymbopogon nardus* essential oils against *P. larvae* have shown an inhibition at 320 mg/L in vitro [68]. Almost all essential oils of *Achyrocline satureioides*, *Chenopodium ambrosioide*, *Eucalyptus cinerea*, *Gnaphalium gaudichaudianum*, *Lippia turbinata*, *Marrubium vulgare*, *Minthostachys verticillata*, *Origanum vulgare*, *Tagetes minuta* and *Thymus vulgaris* were effective against *P. larvae* strains. *Eucalyptus cinerea* and *M. verticillata* essential oils exhibited 100% efficiency in inhibiting the growth of all *P. larvae* strains [69]. Essential oils of *Schinus molle* var. *areira* L., *Acantholippia seriphioides* A. Gray, *Mintosthachys mollis*, *Tagetes minuta* L. and *Lippia turbinata* Griseb grown in wild in Argentina shared minimum and maximum MIC and MBC values of 200–250 mg/L and 200–300 mg/L for Andean thyme and 800–1000 mg/L and 850–1100 mg/L. Andean thyme has been shown to be the most effective in vitro against *P. larvae* and could be a perspective natural alternative to the traditional antimicrobial treatment of AFB pathogen [61].

## 4. Materials and Methods

### 4.1. Samples of Bees

A total of 200 samples of *Apis mellifera carnica* workers were examined. Samples of bees were taken from hives from the eastern Slovakia in the Košice area (48.7164° N, 21.2611° E). Bees were sampled in winter and summer, with samples from the digestive tract (intestines and rectum). examined separately. Workers of honey bees were anesthetized on ice and washed in 86% ethanol before dissection. The head or thorax of a honeybee was fixed and the entire intestine was removed by pulling the stinger using sterile dissecting forceps. The intestines and rectum were separated and collected into sterile, separate microcentrifuge tubes.

The basic dilution (10^−2^) was obtained by homogenizing 0.1 g of the digestive tract contents of five bees and 9.9 mL of peptone saline (0.89%). Selection for groups of microorganisms followed as shown in Table 7. All agars were purchased from Oxoid (Basingstoke, United Kingdom).

### 4.2. Identification of Bacteria

Identification of bacteriota was performed using MALDI-TOF-MS Biotyper (Bruker Daltonics, Bremen, Germany). All the preparatory stages for the samples were carried out according to the MALDI-TOF-MS Biotyper manufacturer’s recommendations. Bacterial colonies were transferred into 300 μL of distilled water and 900 µL of ethanol in Eppendorf tubes, which were centrifuged for 2 min at 14,000 rpm. The supernatant was removed, and centrifugation was repeated for the pellet, which was subsequently allowed to dry. Ten microliters of 70% formic acid and 10 μL of acetonitrile were added to the dried pellet. Tubes were centrifuged for 2 min at 14,000 rpm and 1 μL of the supernatant was applied for identification with the MALDI-TOF. Matrix, α-cyano-4-hydroxycinnamic acid in a volume of 1 μL, was added to that 1 µL of supernatant and allowed to dry. The analysis was performed with a Microflex LT (Bruker Daltonics, Bremen, Germany) instrument and Flex Control 3.4 software and Biotyper Realtime Classification 3.1 with BC specific software. Confidence scores of ≥2.0 and ≥1.7 were the criteria for successful identification at the levels of species and genus, respectively [70].

### 4.3. Antimicrobial Resistance Testing

Antimicrobial susceptibility tests were carried out using the disc diffusion method, whereas the antimicrobial resistance of *Lactobacillus* spp. was assessed using MIC E-tests. Antimicrobial resistance against cefepime (CEF, 30 μg), ciprofloxacin (CIP, 10 μg), ticarcillin (TIC, 10 μg), imipenem (IMI, 10 μg), chloramphenicol (CHL, 10 μg), teicoplanin (TEI, 30 μg), tigecycline (TIG,15 μg), linezolid (LIN, 10 μg), tobramycin (TOB, 10 μg), ampicillin (AMP, 10 μg) or meropenem (MER, 10 μg) (Oxoid, Basingstoke, UK) was examined. Bacteria strains were cultured on Muller Hinton agar for 24 h at 37 °C, suspended in sterile distilled water at approximately 10^5^ cells/mL (A_620_ = 0.388, equivalent to a McFarland standard) and used for testing. The diameters of inhibition zones were measured after incubation. Three replicates were tested for each isolate strain.

For *Lactobacillus* spp. strains, the MICs (μg/mL) of AMP, MER, IMI and CHL were evaluated using the commercial E-test^®^ (Oxoid, Basingstoke, UK). The concentrations of antimicrobials ranged from 0.016 to 256 μg/mL. Bacterial cultures in exponential growth phase were adjusted to a suitable turbidity (10^6^ to 10^7^ CFU/mL) and used for inoculation of iso-sensitized agar (90% *w/v*, Oxoid, UK) supplemented with main Rogosa agar (MRS) or TPY agar (10% *w/v*) (Oxoid, Basingstoke, UK). E-test strips were placed on the surface of the inoculated agar and incubated at 37 °C for 24 h microaerophilically. The MIC test result was interpreted as the point at which the ellipse intersected the E-test strip as described in the E-test technical guide.

### 4.4. Antimicrobial Activity of Bacterial Suspensions against P. larvae

Bacterial strains after 24 h of incubation on MRS and tryptone soya agar (TSA) medium were centrifuged at 5500× *g* for 10 min at 4 °C and 0.1 mL of the supernatant was used for detection of activity against *P. larvae*. A suspension (0.1 mL, 10^5^ CFU/mL) was plated on Mueller–Hinton agar. Filter paper discs (6 mm diameter) were impregnated with 15 μL of supernatant from each bacteria and placed on the *P. larvae*-inoculated agar. The agars were incubated initially at 4 °C for 2 h and then at 37 °C for 16 h. All tests were performed in triplicate. Filter discs impregnated with 10 μL of distilled water were used as a negative control and antibiotics (amikacin, 10 μg and gentamicin, 10 μg) were used as a positive control [71]. Two *P. larvae* isolates were tested in this study: one isolate was from bee hive and second isolate was purchased (*P. larvae* CCM 4483) from the Czech collection of microorganisms (Brno, Czech Republic).

### 4.5. Antimicrobial Activity of Essential Oils against P. larvae

For testing their antimicrobial activity, 30 essential oils purchased from Hanus s.r.o., Slovakia were used in the present study: *Lavandula angustifolia* Mill., *Cinnamomum zeylanicum* L., *Pinus montana* Mill., *Mentha piperita* L., *Foeniculum vulgare* Mill., *Pinus sylvestris* L., *Satureja hortensis* L., *Origanum vulgare* L., *Pimpinella anisum* L., *Rosmarinus officinalis* L., *Salvia officinalis* L., *Abies alba* Mill., *Citrus aurantium* var. *dulce* L., *Citrus sinensis* L. Osbeck., *Cymbopogon nardus* L., *Mentha spicata* var. *crispa* L., *Thymus vulgaris* L., *Carvum carvi* L., *Thymus serpyllum* L., *Amyris balsamifera*, *Ocimum basilicum*, *Canarium luzonicum* Miq., *Eucalyptus globulus*, *Gaultheria procumbens*, *Pelargonium graveolens*, *Cinnamomum caphora* var. *Linalolifera*, *Boswellia carterii*, *Melaleuca leucadendron, Litsea cubeba* Pers. and *Melaleuca ericifolia* Smith. The inoculation and testing technique was as described in Section 4.3.

### 4.6. Statistical Analyses

All measurements were made in triplicate. Statistical processing of data of the bacterial counts was performed using Microsoft Excel^®^ software. Bacterial counts and measurements of inhibition zones were expressed as the means and standard deviation (SD). Student’s *t*-test was used for calculation of significance of variability in distribution of bacteria among seasons as well as among different parts of bee gut for individual groups of analysed microorganisms. Significance of the results was considered at the following thresholds: *p* ≤ 0.05, *p* ≤ 0.01, *p* ≤ 0.001.

## 5. Conclusions

Understanding of bacteriome inhabiting the intestine of bees has a potential to help beekeepers and promote bee health. *Apis mellifera* is the most important pollinator insect in means of global food security. Our studies on characterization and functional role of the bee’s intestinal microbiota reveal the unique properties of *A. mellifera* bacteriota. EU prohibited antibiotics in beekeeping practice and *P. larvae* after antibiotics treatments can develop resistance. Natural antimicrobials as probiotic bacteria and essential oils can play the biggest role in control of bee pathogens.

The antimicrobials may cause an alteration in bee gut microbiota so the studies of beneficiary intestinal bacteria, which may increase colony resistance to various bee’s pathogens, is a promising alternative to bee’s antimicrobial treatment. Essential oils showed the inhibitory effect on *P. larvae* isolated from bees, so the application of essential oils may be expanded in beekeeping. Therefore, the present results on the antimicrobial activity of bee-beneficial bacteria and essential oils from plants can help increase the beekeepers’ awareness of these possibilities and possibly reduce bee colony mortality on a global scale.

## Figures and Tables

**Table 1:** Isolated bacteriota of adult worker honeybee guts in in log cfu/g (mean ± SD).

	Bee Gut from Intestine		Bee Gut from Rectum	
	Winter Bees Samples	Summer Bees Samples	Winter Bees Samples	Summer Bees Samples
TCAM *	5.39 ± 0.14 ^a^	5.03 ± 0.16 ^ab^	5.00 ± 0.22 ^abc^	4.48 ± 0.13 ^abc^
TCANM	8.38 ± 0.11 ^a^	8.12 ± 0.06 ^b^	9.25 ± 0.15 ^ab^	9.05 ± 0.09 ^ab^
AG^+^	6.49 ± 0.13 ^a^	6.13 ± 0.09 ^ab^	7.10 ± 0.12 ^abc^	6.77 ± 0.11 ^abc^
BS	2.48 ± 0.09 ^a^	3.43 ± 0.16 ^ab^	3.53 ± 0.07 ^ac^	3.22 ± 0.10 ^abc^
LS	7.14 ± 0.06 ^a^	7.66 ± 0.14 ^ab^	8.27 ± 0.11 ^ab^	8.12 ± 0.06 ^ab^
PS	2.55 ± 0.06 ^a^	2.29 ± 0.13 ^ab^	3.12 ± 0.07 ^abc^	2.85 ± 0.15 ^abc^
ES	3.21 ± 0.08 ^a^	3.42 ± 0.12 ^ab^	2.24 ± 0.10 ^abc^	2.53 ± 0.15 ^abc^
SS	3.22 ± 0.09 ^a^	3.45 ± 0.08 ^ab^	2.56 ± 0.19 ^abc^	2.25 ± 0.07 ^abc^
CB	2.52 ± 0.11 ^a^	3.25 ± 0.13 ^ab^	3.57 ± 0.13 ^abc^	3.37 ± 0.14 ^ac^

* TCAM—total counts of aerobic microorganisms, TCANM—total counts of anaerobic microorganisms, AG^+^—anaerobic Gram-positive bacteria, BS—*Bacillus* spp., LS—*Lactobacillus* spp., PS—*Pseudomonas* spp., ES—*Enterococcus* spp., SS—*Staphylococcus* spp., CB—coliform bacteria. ^a,b,c^ same letters in the raw show statistically significant differences among the groups.

**Table 2:** Isolated species of adult worker honeybee bacteriota from gastrointestinal tract.

Class	Genus	Species
Gammaproteobacteria	*Aeromonas*	*Aeromonas salmonicida*
Actinobacteria	*Arthrobacter*	*Arthrobacter tumbae*
Firmicutes	*Bacillus*	*Bacillus cereus*
Firmicutes	*Bacillus*	*Bacillus circulans*
Firmicutes	*Bacillus*	*Bacillus licheniformis*
Firmicutes	*Bacillus*	*Bacillus megaterium*
Firmicutes	*Bacillus*	*Bacillus oleronius*
Firmicutes	*Bacillus*	*Bacillus* spp.
Firmicutes	*Bacillus*	*Bacillus subtilis*
Firmicutes	*Bacillus*	*Bacillus thuringiensis*
Gammaproteobacteria	*Citrobacter*	*Citrobacter* spp.
Gammaproteobacteria	*Citrobacter*	*Citrobacter braakii*
Gammaproteobacteria	*Citrobacter*	*Citrobacter koseri*
Betaproteobacteria	*Delftia*	*Delftia acidovorans*
Gammaproteobacteria	*Enterobacter*	*Enterobacter aerogenes*
Gammaproteobacteria	*Enterobacter*	*Enterobacter clocae*
Gammaproteobacteria	*Enterobacter*	*Enterobacter kobei*
Firmicutes	*Enterococcus*	*Enterococcus cloacae*
Firmicutes	*Enterococcus*	*Enterococcus faecalis*
Gammaproteobacteria	*Escherichia*	*Escherichia coli*
Firmicutes	*Fructobacillus*	*Fructobacillus fructosus*
Gammaproteobacteria	*Hafnia*	*Hafnia alvei*
Gammaproteobacteria	*Klebsiella*	*Klebsiella aerogenes*
Gammaproteobacteria	*Klebsiella*	*Klebsiella oxytoca*
Gammaproteobacteria	*Klebsiella*	*Klebsiella pneumoniae*
Gammaproteobacteria	*Klebsiella*	*Klebsiella variicola*
Actinobacteria	*Kocuria*	*Kocuria kristinae*
Firmicutes	*Lactobacillus*	*Lactobacillus acidophilus*
Firmicutes	*Lactobacillus*	*Lactobacillus agilis*
Firmicutes	*Lactobacillus*	*Lactobacillus apis*
Firmicutes	*Lactobacillus*	*Lactobacillus brevis*
Firmicutes	*Lactobacillus*	*Lactobacillus crispatus*
Firmicutes	*Lactobacillus*	*Lactobacillus jensenii*
Firmicutes	*Lactobacillus*	*Lactobacillus kunkeei*
Firmicutes	*Lactobacillus*	*Lactobacillus mellis*
Firmicutes	*Lactobacillus*	*Lactobacillus plantarum*
Firmicutes	*Lactobacillus*	*Lactobacillus* spp.
Firmicutes	*Lactococcus*	*Lactococcus garvieae*
Firmicutes	*Lactococcus*	*Lactococcus lactis*
Actinobacteria	*Microbacterium*	*Microbacterium pumilum*
Actinobacteria	*Microbacterium*	*Microbacterium testaceum*
Gammaproteobacteria	*Moraxella*	*Moraxella* spp.
Gammaproteobacteria	*Morganella*	*Morganella morgani*
Firmicutes	*Paenibacillus*	*Paenibacillus larvae*
Gammaproteobacteria	*Pantotea*	*Pantotea agglomerans*
Gammaproteobacteria	*Pantotea*	*Pantotea ananatis*
Gammaproteobacteria	*Pantotea*	*Pantotea vagans*
Gammaproteobacteria	*Proteus*	*Proteus mirabilis*
Gammaproteobacteria	*Pseudomonas*	*Pseudomonas marginalis*
Gammaproteobacteria	*Pseudomonas*	*Pseudomonas oryzihabitans*
Gammaproteobacteria	*Pseudomonas*	*Pseudomonas putida*
Gammaproteobacteria	*Rahnella*	*Rahnella aquatilis*
Gammaproteobacteria	*Rahnella*	*Rahnella terrigena*
Betaproteobacteria	*Ralstonia*	*Ralstonia picketii*
Gammaproteobacteria	*Raoultella*	*Raoultella ornithinolytica*
Gammaproteobacteria	*Raoultella*	*Raoultella planticola*
Gammaproteobacteria	*Serratia*	*Serratia fonticola*
Gammaproteobacteria	*Serratia*	*Serratia liquefaciens*
Gammaproteobacteria	*Serratia*	*Serratia marcescens*
Alphaproteobacteria	*Sphingomonas*	*Sphingomonas parapaucimobilis*
Alphaproteobacteria	*Sphingomonas*	*Sphingomonas melonis*
Firmicutes	*Staphylococcus*	*Staphylococcus capitis*
Firmicutes	*Staphylococcus*	*Staphylococcus epidermidis*
Firmicutes	*Staphylococcus*	*Staphylococcus hemolyticus*
Firmicutes	*Staphylococcus*	*Staphylococcus hominis*
Firmicutes	*Staphylococcus*	*Staphylococcus warneri*

**Table 3:** Frequency of isolated bacteriota (%) detected in the samples of bee digestive tract.

Species	No. of Isolates/No. of Samples	No. of Positive Samples (%)
*Aeromonas salmonicida*	15/12	6.00
*Arthrobacter tumbae*	21/15	7.50
*Bacillus cereus*	51/25	12.50
*Bacillus circulans*	35/10	5.00
*Bacillus licheniformis*	64/25	12.50
*Bacillus megaterium*	128/96	48.00
*Bacillus oleronius*	25/20	10.00
*Bacillus* spp.	125/52	26.00
*Bacillus subtilis*	56/35	17.50
*Bacillus thuringiensis*	68/42	21.00
*Citrobacter* spp.	188/112	56.00
*Citrobacter braakii*	37/15	7.50
*Citrobacter koseri*	60/30	15.00
*Delftia acidovorans*	150/200	100.00
*Enterobacter aerogenes*	136/110	55.00
*Enterobacter clocae*	126/99	49.50
*Enterobacter kobei*	59/32	16.00
*Enterococcus cloacae*	56/15	7.50
*Enterococcus faecalis*	150/100	50.00
*Escherichia coli*	350/200	100.00
*Fructobacillus fructosus*	29/11	5.50
*Hafnia alvei*	218/169	84.50
*Klebsiella aerogenes*	59/28	14.00
*Klebsiella oxytoca*	98/58	29.00
*Klebsiella pneumoniae*	36/12	6.00
*Klebsiella variicola*	45/15	7.50
*Kocuria kristinae*	186/125	62.50
*Lactobacillus acidophilus*	64/30	15.00
*Lactobacillus agilis*	55/20	10.00
*Lactobacillus apis*	123/69	34.50
*Lactobacillus brevis*	150//100	50.00
*Lactobacillus crispatus*	164//88	44.00
*Lactobacillus jensenii*	15/10	5.00
*Lactobacillus kunkeei*	135/120	60.00
*Lactobacillus mellis*	64/35	17.50
*Lactobacillus plantarum*	95/80	40.00
*Lactobacillus* spp.	167/150	75.00
*Lactococcus garvieae*	121/90	45.00
*Lactococcus lactis*	68/39	19.50
*Microbacterium pumilum*	15/5	2.50
*Microbacterium testaceum*	25/10	5.00
*Moraxella* spp.	55/15	7.50
*Morganella morgani*	115/100	50.00
*Paenibacillus larvae*	1/1	0.50
*Pantotea agglomerans*	52/40	20.00
*Pantotea ananatis*	65/30	15.00
*Pantotea vagans*	87/58	29.00
*Proteus mirabilis*	120/95	47.50
*Pseudomonas marginalis*	12/3	1.50
*Pseudomonas oryzihabitans*	65/50	25.00
*Pseudomonas putida*	35/15	7.50
*Rahnella aquatilis*	65/40	20.00
*Rahnella terrigena*	35/22	11.00
*Ralstonia picketii*	126/110	55.00
*Raoultella ornithinolytica*	69/52	26.00
*Raoultella planticola*	35/15	7.50
*Serratia fonticola*	95/95	47.50
*Serratia liquefaciens*	87/58	29.00
*Serratia marcescens*	64/30	15.00
*Sphingomonas parapaucimobilis*	125/100	50.00
*Sphingomonas melonis*	120/60	30.00
*Staphylococcus capitis*	136/120	60.00
*Staphylococcus epidermidis*	168/62	31.00
*Staphylococcus haemolyticus*	58/35	17.50
*Staphylococcus hominis*	112/90	45.00
*Staphylococcus warneri*	64/52	26.00

**Table 4:** Antimicrobial resistance of bacteria isolated from bee digestive tracts.

Antimicrobial	CEF	CIP	–	–
Resistance/Sensitivity	R/S	R/S	–	–
*Aeromonas salmonicida*	0/15	0/15	–	–
*Arthrobacter tumbae*	ND	ND	–	–
*Bacillus cereus*	ND	ND	–	–
*Bacillus circulans*	ND	ND	–	–
*Bacillus licheniformis*	ND	ND	–	–
*Bacillus megaterium*	ND	ND	–	–
*Bacillus oleronius*	ND	ND	–	–
*Bacillus* spp.	ND	ND	–	–
*Bacillus subtilis*	ND	ND	–	–
*Bacillus thuringiensis*	ND	ND	–	–
	TIC	IMI	CIP	CHL
	R/S	R/S	R/S	R/S
*Citrobacter* spp.	8/188	25/188	0/188	45/188
*Citrobacter braakii*	6/37	15/37	5/37	10/37
*Citrobacter koseri*	16/60	10/60	5/60	14/60
*Delftia acidovorans*	ND	ND	ND	ND
	TIC	IMI	CIP	CHL
	R/S	R/S	R/S	R/S
*Enterobacter aerogenes*	61/136	25/136	10/136	22/136
*Enterobacter clocae*	28/126	5/136	1/136	6/136
*Enterobacter kobei*	9/59	5/59	0/59	0/59
	IMI	TEI	TIG	–
	R/S	R/S	R/S	–
*Enterococcus cloacae*	5/56	6/56	11/56	–
*Enterococcus faecalis*	58/150	10/150	25/150	–
	TIC	IMI	CIP	CHL
	R/S	R/S	R/S	R/S
*Escherichia coli*	53/350	26/350	12/350	10/350
*Fructobacillus fructosus*	ND	ND	ND	ND
*Hafnia alvei*	15/218	12/218	5/218	5/218
*Klebsiella aerogenes*	42/59	25/59	15/59	5/59
*Klebsiella oxytoca*	63/98	35/98	15/98	10/98
*Klebsiella pneumoniae*	14/36	10/36	5/36	1/36
*Klebsiella variicola*	5/45	10/45	4/45	5/45
*Kocuria kristinae*	ND	ND	ND	ND
	AMP	IMI	MER	CHL
	R/S	R/S	R/S	R/S
*Lactobacillus acidophilus*	4/64	0/64	0/64	0/64
*Lactobacillus agilis*	2/55	3/55	2/55	0/55
*Lactobacillus apis*	16/123	10/123	8/123	5/123
*Lactobacillus brevis*	15/150	20/150	10/150	15/150
*Lactobacillus crispatus*	25/164	38/164	5/164	6/164
*Lactobacillus jensenii*	0/15	0/15	0/15	0/15
*Lactobacillus kunkeei*	52/135	25/135	15/135	10/135
*Lactobacillus mellis*	2/64	0/64	1/64	0/64
*Lactobacillus plantarum*	50/95	20/95	10/95	10/95
*Lactobacillus* spp.	0/167	0/167	0/167	0/167
*Lactococcus garvieae*	ND	ND	ND	ND
*Lactococcus lactis*	ND	ND	ND	ND
*Microbacterium pumilum*	ND	ND	ND	ND
*Microbacterium testaceum*	ND	ND	ND	ND
*Moraxella* spp.	ND	ND	ND	ND
	TIC	IMI	CIP	CHL
	R/S	R/S	R/S	R/S
*Morganella morgani*	65/115	35/115	25/115	15/115
*Paenibacillus larvae*	ND	ND	ND	ND
*Pantotea agglomerans*	15/52	15/52	10/52	10/52
*Pantotea ananatis*	10/65	15/65	15/65	10/65
*Pantotea vagans*	37/87	30/87	15/87	10/87
*Proteus mirabilis*	25/120	15/120	16/120	10/120
	TIC	IMI	CIP	TOB
	R/S	R/S	R/S	R/S
*Pseudomonas marginalis*	5/12	4/12	2/12	0/12
*Pseudomonas oryzihabitans*	30/65	20/65	10/65	10/65
*Pseudomonas putida*	5/35	5/35	5/35	5/35
	TIC	IMI	CIP	CHL
	R/S	R/S	R/S	R/S
*Rahnella aquatilis*	24/65	20/65	12/65	8/65
*Rahnella terrigena*	5/35	0/35	0/35	0/35
*Ralstonia picketii*	ND	ND	ND	ND
*Raoultella ornithinolytica*	29/69	20/69	10/69	10/69
*Raoultella planticola*	15/35	20/35	10/35	5/35
*Serratia fonticola*	45/95	30/95	15/95	5/95
*Serratia liquefaciens*	25/87	32/87	16/87	10/87
*Serratia marcescens*	16/64	12/64	5/64	2/64
*Sphingomonas parapaucimobilis*	ND	ND	ND	ND
*Sphingomonas melonis*	ND	ND	ND	ND
	TIG	LIN	CIP	CHL
	R/S	R/S	R/S	R/S
*Staphylococcus capitis*	15/136	25/136	20/136	10/136
*Staphylococcus epidermidis*	60/168	30/168	15/168	5/168
*Staphylococcus haemolyticus*	28/58	15/58	10/58	5/58
*Staphylococcus hominis*	41/112	23/112	16/112	7/112
*Staphylococcus warneri*	5/64	15/64	10/64	5/64

CEF—cefepime; CIP—ciprofloxacin; TIC—ticarcillin; IMI—imipenem; CHL—chloramphenicol; TEI—teicoplanin; TIG—tigecycline; LIN—linezolid; TOB—tobramycin; AMP—ampicillin; MER–meropenem. ND—not defined. R– resistant; S—sensitive.

**Table 5:** Antimicrobial activity of individual isolates against *P. larvae* in mm (mean ± SD of three replicates).

Species	*P. larvae*	*P. larvae* CCM 4483
*Aeromonas salmonicida*	10.67 ± 0.58	10.33 ± 0.58
*Arthrobacter tumbae*	9.67 ± 1.15	8.67 ± 0.58
*Bacillus cereus*	14.33 ± 0.58	13.67 ± 0.58
*Bacillus circulans*	14.67 ± 1.15	14.33 ± 0.58
*Bacillus licheniformis*	15.67 ± 0.58	16.33 ± 1.15
*Bacillus megaterium*	11.67 ± 0.58	11.33 ± 0.58
*Bacillus oleronius*	10.33 ± 1.15	10.67 ± 0.58
*Bacillus* spp.	9.33 ± 0.58	8.67 ± 0.58
*Bacillus subtilis*	12.33 ± 0.58	11.67 ± 0.58
*Bacillus thuringiensis*	12.33 ± 1.15	11.67 ± 1.15
*Citrobacter* spp.	8.67 ± 0.58	6.67 ± 1.53
*Citrobacter braakii*	8.33 ± 1.53	7.33 ± 1.15
*Citrobacter koseri*	6.33 ± 1.53	7.67 ± 0.58
*Delftia acidovorans*	11.67 ± 1.15	11.33 ± 0.58
*Enterobacter aerogenes*	8.67 ± 0.58	6.67 ± 1.53
*Enterobacter clocae*	8.33 ± 1.53	7.33 ± 1.15
*Enterobacter kobei*	6.33 ± 1.53	7.67 ± 0.58
*Enterococcus cloacae*	14.67 ± 0.58	14.33 ± 0.58
*Enterococcus faecalis*	16.33 ± 1.53	16.33 ± 0.58
*Escherichia coli*	15.67 ± 0.58	15.33 ± 0.58
*Fructobacillus fructosus*	18.67 ± 0.58	18.33 ± 0.58
*Hafnia alvei*	8.33 ± 1.53	7.33 ± 1.15
*Klebsiella aerogenes*	6.33 ± 1.53	7.67 ± 0.58
*Klebsiella oxytoca*	7.67 ± 0.58	8.33 ± 0.58
*Klebsiella pneumoniae*	7.33 ± 0.58	6.67 ± 0.58
*Klebsiella variicola*	5.33 ± 0.58	4.67 ± 0.58
*Kocuria kristinae*	11.33 ± 0.58	10.67 ± 0.58
*Lactobacillus acidophilus*	23.33 ± 0.58	22.67 ± 0.58
*Lactobacillus agilis*	18.67 ± 0.58	18.33 ± 0.58
*Lactobacillus apis*	20.33 ± 0.58	20.67 ± 0.58
*Lactobacillus brevis*	19.33 ± 0.58	19.00 ± 1.00
*Lactobacillus crispatus*	20.33 ± 1.15	19.67 ± 1.15
*Lactobacillus jensenii*	20.33 ± 0.58	20.33 ± 1.15
*Lactobacillus kunkeei*	25.67 ± 1.15	24.33 ± 0.58
*Lactobacillus mellis*	18.67 ± 1.15	17.67 ± 0.58
*Lactobacillus plantarum*	22.33 ± 0.58	21.67 ± 0.58
*Lactobacillus* spp.	17.00 ± 1.00	17.33 ± 0.58
*Lactococcus garvieae*	16.67 ± 0.58	16.33 ± 0.58
*Lactococcus lactis*	17.67 ± 0.58	17.33 ± 0.58
*Microbacterium pumilum*	13.67 ± 0.58	13.33 ± 0.58
*Microbacterium testaceum*	12.67 ± 0.58	12.33 ± 0.58
*Moraxella* spp.	8.67 ± 0.58	6.67 ± 1.53
*Morganella morgani*	8.33 ± 1.53	7.33 ± 1.15
*Pantotea agglomerans*	6.33 ± 1.53	7.67 ± 0.58
*Pantotea ananatis*	8.67 ± 0.58	6.67 ± 1.53
*Proteus mirabilis*	8.33 ± 1.53	7.33 ± 1.15
*Pantotea vagans*	6.33 ± 1.53	7.67 ± 0.58
*Pseudomonas marginalis*	11.33 ± 0.58	10.67 ± 0.58
*Pseudomonas oryzihabitans*	11.33 ± 1.15	11.00 ± 1.00
*Pseudomonas putida*	10.67 ± 0.58	10.33 ± 0.58
*Rahnella aquatilis*	8.67 ± 0.58	6.67 ± 1.53
*Rahnella terrigena*	8.33 ± 1.53	7.33 ± 1.15
*Ralstonia picketii*	6.33 ± 1.53	7.67 ± 0.58
*Raoultella ornithinolytica*	8.67 ± 0.58	6.67 ± 1.53
*Raoultella planticola*	8.33 ± 1.53	7.33 ± 1.15
*Serratia fonticola*	8.67 ± 0.58	6.67 ± 1.53
*Serratia liquefaciens*	8.33 ± 1.53	7.33 ± 1.15
*Serratia marcescens*	6.33 ± 1.53	7.67 ± 0.58
*Sphingomonas parapaucimobilis*	11.67 ± 1.15	11.33 ± 0.58
*Sphingomonas melonis*	10.67 ± 0.58	10.33 ± 0.58
*Staphylococcus capitis*	13.67 ± 0.58	13.33 ± 0.58
*Staphylococcus epidermidis*	14.67 ± 0.58	14.33 ± 0.58
*Staphylococcus haemolyticus*	13.67 ± 0.58	13.33 ± 0,58
*Staphylococcus hominis*	12.67 ± 0.58	12.33 ± 0.58
*Staphylococcus warneri*	11.67 ± 0.58	11.33 ± 0.58

**Table 6:** Antimicrobial activity of essential oils against *P. larvae* in mm.

Essential Oil	*P. larvae*	*P. larvae* CCM 4483
*Lavandula angustifolia* Mill.	14.33 ± 1.15	15.33 ± 0.58
*Cinnamomum zeylanicum* L.	10.00 ± 1.00	12.33 ± 2.52
*Pinus montana* Mill.	17.67 ± 0.58	17.33 ± 0.58
*Mentha piperita L.*	7.33 ± 0.58	7.00 ± 2.00
*Foeniculum vulgare* Mill.	14.66 ± 0.58	14.00 ± 0.57
*Pinus sylvestris* L.	17.00 ± 1.00	17.67 ± 0.57
*Satureja hortensis L.*	12.33 ± 0.58	17.67 ± 1.53
*Origanum vulgare* L.	18.67 ± 1.15	19.00 ± 1.00
*Pimpinella anisum* L.	12.33 ± 0.58	11.67 ± 0.58
*Rosmarinus officinalis* L.	14.67 ± 0.58	10.00 ± 1.00
*Salvia officinalis* L.	14.33 ± 0.58	13.00 ± 1.00
*Abies alba* Mill.	17.33 ± 0.58	18.00 ± 1.00
*Citrus aurantium* var. *dulce* L.	4.33 ± 0.58	3.00 ± 1.00
*Citrus sinensis* L. Osbeck.	2.00 ± 1.00	5.33 ± 0.58
*Cymbopogon nardus* L.	8.67 ± 0.58	8.00 ± 1.00
*Mentha spicata* var. *crispa* L.	9.67 ± 1.53	9.33 ± 0.57
*Thymus vulgaris* L.	19.67 ± 1.53	15.67 ± 1.53
*Carvum carvi* L.	7.67 ± 0.58	5.00 ± 0.58
*Thymus serpyllum* L.	4.33 ± 0.58	7.33 ± 0.58
*Amyris balsamifera*	9.33 ± 0.58	9.67 ± 0.58
*Ocimum basilicum*	13.67 ± 1.15	14.00 ± 1.00
*Canarium luzonicum* Miq.	11.33 ± 1.15	12.33 ± 0.58
*Eucalyptus globulus*	16.33 ± 1.15	17.33 ± 0.58
*Gaultheria procumbens*	8.33 ± 0.58	7.33 ± 0.58
*Pelargonium graveolens*	6.67 ± 0.58	7.33 ± 0.58
*Cinnamomum caphora* var. *linalolifera*	16.00 ± 1.73	15.67 ± 1.15
*Boswellia carterii*	7.67 ± 1.15	7.00 ± 1.00
*Melaleuca leucadendron*	9.67 ± 0.58	9.33 ± 0.58
*Litsea cubeba* Pers.	10.33 ± 0.58	10.66 ± 0.58
*Melaleuca ericifolia* Smith.	9.67 ± 0.58	10.00 ± 1.00

**Table 7:** Incubation conditions of bacteriota of the intestine of honey bees.

Microorganisms Group	Dilution	Agar	Inoculation Method	Cultivation Condition		
				Relation of O_2_	Temperature	Time
TCAM	10^−5^–10^−7^	PCA	surface	aerobic	30 °C	48 h
TCANM	10^−5^–10^−7^	PCA	surface	anaerobic	25 °C	48 h
AG^+^	10^−3^–10^−6^	AA	surface	anaerobic	37 °C	48 h
*Bacillus* spp.	10^−3^–10^−5^	PCA	surface	aerobic	30 °C	48 h
*Lactobacillus* spp.	10^−2^–10^−6^	MRS	surface	aerobic	37 °C	48 h
*Pseudomonas* spp.	10^−3^–10^−5^	*Pseudomonas* agar	surface	aerobic	30 °C	48 h
*Enterococcus* spp.	10^−3^–10^−5^	*Enterococcus* selective agar	surface	aerobic	37 °C	48 h
*Staphylococcus* spp.	10^−2^–10^−4^	Blood agar	surface	aerobic	37 °C	48 h
CB	10^−4^–10^−6^	McC	surface	aerobic	37 °C	48 h

TCAM—total counts of aerobic microorganisms; TCNANM—total counts of anaerobic microorganisms; AG^+^—anaerobic Gram-positive bacteria; CB—coliform bacteria; PCA—plate count agar; AA—anaerobic agar; MRS—Main Rogosa agar; McC—MacConkey agar.
